# Development of molecular method for sex identification in date palm (*Phoenix dactylifera* L.) plantlets using novel sex-linked microsatellite markers

**DOI:** 10.1007/s13205-015-0321-6

**Published:** 2016-01-09

**Authors:** Maryam, Muhammad Jafar Jaskani, Faisal Saeed Awan, Saeed Ahmad, Iqrar A. Khan

**Affiliations:** 1 Institute of Horticultural Sciences, University of Agriculture Faisalabad, Faisalabad, 38000 Pakistan; 2 Centre of Agricultural Biochemistry and Biotechnology, University of Agriculture, Faisalabad, 38000 Pakistan

**Keywords:** *Phoenix dactylifera* L., SSR marker, Sex identification, Polymorphism

## Abstract

Microsatellite markers containing simple sequence repeats (SSRs) are a valuable tool for genetic analysis. Date palm is a dioecious and slow flowering and is very difficult to identify the gender of the trees until it reaches the reproductive age (5–10 years). A total of 12 microsatellite primers were used with 30 date palm samples, 14 parents (8 male + 6 females) and 16 progeny (developed from parents breeding) which showed that microsatellites were highly polymorphic, having a great number of alleles. A total of 124 alleles were characterized in 12 SSR loci. On average, there are 9.08 alleles per locus, with a range from 5 to 16 alleles, for primers mpdCIR15 and mpdCIR57, respectively. These primers produced 15 polymorphic loci specifically in male date palm samples and the seedlings harboring the unique fragments were further characterized as male plants. Increasingly, 38.46 % of these loci were scored as homozygous alleles while 61.53 % heterozygous allelic loci were determined. Primer mpdCIR48 produced a specific locus (250/250) in all male samples whereas the same locus was absent in female samples. Similarly, a locus of 300/310 bp reoccurred in 5 date palm male samples using marker DP-168 which indicated that these are the promising candidate marker to detect the sex in date palm seedlings at early stage. The data resulted from combination of 12 primers enabled the 16 seedling samples progeny (developed from parents breeding) of date palm cultivars to divide into two groups i.e., male and female regarding their sex expression comparative to the parents (male + female) using the principle coordinate analysis.

## Introduction

Date palm (2*n* = 36) is dioecious monocotyledonous belonging to Arecaceae family with 200 genera and 500 species (Dowson 1982). Phoenix is an important genus in the family which is most captivating (Munier 1973) as they exert great variation in their reproductive morphology, where single sex flowers are present on more than 85 % palms (Dransfield et al. 2008). In Pakistan, it is the 3rd major fruit crop after citrus and mango, and about 325 varieties of date palm are reported (Botes and Zaid 2002).

Sex determination at early stages of growth is very important to facilitate the breeding programs. It is hard to differentiate the date palm sex at early stages, so one cannot employ the genetic diversity programs until it reached reproductive stage (5–10 years) (Bendiab et al. 1993). As date palm is dioecious, sex chromosomes are observed in homomorphic form. An extra-heterochromatin present on the both arms of the male chromosomes is considered to be sex determinant (Siljak-Yakovlev et al. 1996). Historically, an improved breeding program is lacking due to presence of low genetic diversity as simple and perfect method for gender differentiation before first flowering was absent (Aberlenc-Bertossi et al. 2011). Date palm progenies contain equal proportion of male and female plants which has been directed to the hypothesis that sex is found genetically (Daher et al. 2010). The sex chromosomes existence in date palm on the basis of cytological studies was proposed with chromomycin staining, however, the genes associated with sex have not been determined Siljak-Yakovlev et al. (1996). Similarly, the process of developmental stages that arrest the sterile sex organs has not been studied in detail. Using cytological and biochemical means, they concluded that there is no significant difference between male and female individuals and genetically its an important task to discriminate the sex agronomically in monoecious and dioecious species due to the longevity. Efforts have been made to understand the basic requirement of gender determination at early stage and use of the isozymes analysis (Torres and Tisserat 1980), peroxidases (Majourhat et al. 2002) and molecular marker tools with random amplified polymorphic DNA (RAPD) (Moghaieb et al. 2010).

Biochemical studies give in a little information for sex identification of immature plants and gender specific DNA sequences offer a more capable and efficient method for gender determination (Qacif et al. 2007). Various efforts had been made to comprehend the genetic basis of sex evaluation of plants at early stages of development with molecular tools (Biffi et al. 1995; Hormaza et al. 1994). *Asparagus*, *Mercurialis*, *Vitis* and *Spinacia* are the well-documented examples in which sex identification was hindered due to the existence of additional factors and alleles that change the ultimate effect of sex determining genes (Durand and Durand 1990).

The recent techniques offer a new tool for genetic characterization and linkage maps construction with the use of polymerase chain reaction (PCR). 28,889 EST sequences from the date palm genome database were analyzed to identify simplesequence repeats (SSRs) and to develop gene-based markers, i.e. expressed sequence tag-SSRs (EST-SSRs) (Zhao et al. 2013).

Arbitrary primers were used for the DNA template amplification in random amplified polymorphic DNA (RAPD) technique (Welsh and McClelland 1990). Very effective studies have been made on numerous plant species for linkage and evolution studies (Halward et al. 1992; Carlson et al. 1991). RFLPs and RAPD markers are effectively used for molecular studies of date palm (Abdallah et al. 2000; Trifi et al. 2000) to discriminate the sex-specific DNA with different molecular techniques (RAPD and ISSR) for the collection and reformation of outstanding male pollen. Molecular breeding would accelerate genetic improvement of fruit tree through marker-assisted selection. However, the lack of molecular markers in date palm restricts the application of molecular breeding.

In this study, we have identified the sex-specific DNA markers for date palm cultivars using microsatellites. Such a technique will not only facilitate the screening of gender at early developmental stage but also could be most important for speeding up breeding and, thereby, saving of time, cost and other resources.

## Materials and methods

### Plant material and genomic DNA extraction

Leaf samples of eight male and six female date palm trees were collected from two different locations of Horticultural Fruit Garden Square No. 9 and Square No 32 and Dera Ismail Khan while the leaves of 1-year-old progeny developed from hybridization of these parents were collected from the fruit plant nursery, University of Agriculture, Faisalabad, Pakistan (Table 3). The frozen young leaves were cleaned carefully with the distilled water to remove the waxy layer. Gene JET Plant Genomic DNA Purification Mini Kit was used to extract the DNA by following the manual instructions of the kit (Thermo Scientific). Quality of DNA was assessed by electrophoresis on 0.8 % agarose gel and its quality and quantity was evaluated in Nanodrop ND-1000 spectrophotometer (Nanodrop Technologies, Wilmington, Dalware) to dilute DNA stock as 15 ng/µL d_3_H_2_O to use in PCR amplification.

### Microsatellite amplification

Polymerase chain reaction (PCR) was carried out using 20 µL of reaction mixture containing 2 µL (10 ng) of total genomic DNA, 2 µL of 10× buffer solution [(NH_4_)_4_SO_4_]Mg^+^, 6.4 µL of dNTPs (5 mM), 2.0 µL of MgCl_2_ (25 mM), Primer (Forward and Reverse) 10× from 100× (1.0 µL for each), Taq DNA polymerase 0.2 and 5.8 µL of nuclease-free water. Amplification was carried out in veriti 96-well fast thermal cycler (Peq Lab) under the following conditions: initial DNA denaturation at 95 °C for 10 min, 35 cycles (denaturation at 95 °C for 30 s, annealing temperature depending upon the primer for 30 s and extension at 72 °C for 1 min) and final extension at 72 °C for 7 min. Resolution of PCR products of 30 date palm parents and progeny amplified by 12 SSR primers were visualized by 6 % denaturing polyacrylamide (PAGE) gel followed by ethidium bromide.

### Statistical analysis

The bands of DNA fragments on SSR analysis were scored in a (0–1) binary format (0 for absence, 1 for presence) for allele(s) on respective locus. Efficacy and degree of polymorphism of reported SSR markers were assessed through power marker (Liu and Muse 2004) and principle component analysis for assessing genetic diversity between parents and progeny accessions was performed in PAST (Hammer and Khoshbakht 2005).

## Results and discussion

The 12 primers examined in this study effectively generated clearly amplified SSR bands with different sizes ranging from 100 bp with primer mpdCIR16 to 500 bp with primer mpdCIR32 and mpdCIR57 (Table 1). The different band size range was reported by some other scientists like Ahmed and Al-Qaradawi (2009) and Zehdi et al. (2004b) who amplified the band size ranging from 100 to 300 bp and 173 to 318 bp, respectively. Total 109 alleles were scored with a mean of 9.08 alleles per locus while allelic range varied from 5 to 16 with primers mpdCIR15 and mpdCIR57 (Table 1), whereas Elmeer and Mattat (2012) reported 3–13 alleles and average 4 alleles per locus was identified by Ahmad and Al-Qaradawi (2009). In this study, more number of alleles appeared than previous studies which showed more polymorphism in Pakistani date palm cultivars; however, huge difference of Sudan cultivars may be due to difference of genotype in addition to more number of microsatellites (Durand and Durand 1990). Genetic diversity varied from 0.38 to 0.58 with a mean value 0.47 that is less than Sudanese date palm germplasm (0.70) Zehdi et al. (2004a) and Tunisian date palm, i.e. 0.853 (Elshibli and Korpelainen 2007). As all the progenies developed by hybridization, it is supposed that they shared common genetic basis. However, some progeny diverged from others due to mutational events that might occur during selection.

**Table 1:** SSR primers detail and summary of genetic diversity information by locus in date palm parents and progeny cvs. Hillawi and Khudrawy

Sr. no	PC	AS	MAF	GN	AN	GD	HZ	PIC
1	mPdCIR010	145–340	0.51	18	15	0.58	0.31	0.50
2	mPdCIR015	140–190	0.5	4	5	0.54	0.73	0.46
3	mPdCIR016	100–220	0.55	10	9	0.51	0.33	0.40
4	mPdCIR025	210–320	0.62	10	8	0.48	0.36	0.42
5	mPdCIR032	300–500	0.67	9	9	0.43	0.4	0.34
6	mPdCIR035	190–275	0.69	8	8	0.42	0.24	0.36
7	mPdCIR048	125–200	0.74	5	5	0.39	0.28	0.33
8	mPdCIR057	80–500	0.58	17	16	0.5	0.38	0.42
9	mPdCIR070	150–410	0.65	13	10	0.45	0.38	0.38
10	mPdCIR085	180–400	0.55	8	7	0.51	0.66	0.40
11	mPdCIR093	75–240	0.7	9	9	0.38	0.35	0.32
12	DP168	190–300	0.74	7	8	0.4	0.28	0.34
Total			7.5	118	109	5.59	4.7	4.7
Mean			0.63	9.83	9.08	0.47	0.39	0.39

*PC* primer code, *AS* allele size, *MAF* major allele frequency, *GN* genotype number, *AN* allele number, *GD* genetic diversity, *HZ* heterozygosity, *PIC* polymorphic information contents

Sex determination is a fundamental and significant developmental process in the plant life cycle of all sexually reproducing plants and is economically very important. Sexual phenotypes in commercial crops dictate the method of cultivation and breeding. Long juvenile phase is an obstacle in date palm breeding progenies. Molecular tools have the promises to help out in discriminating male and female seedlings at their early growth stages.

All primers yielded the specific loci in male and female plants separately. These primers produced 15 polymorphic loci specifically in male date palm samples and the same DNA fragments were identified in date palm seedlings (Table 2), and the seedlings harboring the unique fragments were further characterized as male plants. Increasingly, 38.46 % of these loci were scored as homozygous alleles (Table 2), while 61.53 % were determined as heterozygous allelic loci. Male-6 was detected with minimum number of markers, only two markers identified this male; however maximum in male-4 where 10 markers out of 12 detected the male characters (Table 2). Microsatellite primers used in this study for sex identification at seedling stage were highly polymorphic and generated high number of alleles that confirmed the good transferability Billotte et al. (2004). Some primers had problematic loci that have already been reported for erratic amplification (Billotte et al. 2004; Ahmed and Al-Qaradawi 2009) and were excluded from the study, i.e. mPdCIR44 and mPdCIR78. In previous studies, mPdCIR48 did not amplify (Zehdi et al. 2004a, b; Henderson 2009) but in the present study this primer amplified successfully with low magnification.

**Table 2:** Homozygous and heterozygous allele (base pairs) markers which identified male date palm seedlings

Sr. no.	Markers	M1	M2	M3	M4	M5	M6	M7	M8
1	CIR10		230/230	335/340	230/240	230/240			
2	CIR15			160/170	160/170	160/170		170/170	
3	CIR16	190/190		180/180					
4	CIR25	270/270	270/270		270/270	270/270		270/270	270/270
5	CIR32		290/294		290/294			290/294	
6	CIR35				240/235	240/235		240/235	235/235
7	CIR48	250/250	245/250	245/250	245/245	245/250	245/250	245/250	245/250
8	CIR57		295/295	80/85	80/85				
9	CIR70				300/310				300/310
10	CIR85				160/162				160/162
11	CIR93	170/175				170/175		170/175	
12	DP168		300/310	300/310	300/310	300/310	300/310		

In agriculturally important plants like date palm, kiwi fruit, pistachio and papaya, female trees produce the commercial crop while in asparagus, better quality harvest is obtained from male plants. So identification of such type of plants at early developmental stage is of great importance. Moreover, studies on dioecy through marker technology could provide better understanding for development (Ainsworth et al. 1998) and evolutionary pathways of dimorphism (Charlesworth and Charlesworth 1978; Charlesworth 1996).

Total 19 loci were scored from 12 primers which identified the male progeny and similarly 22 loci were recorded only in date palm using 14 primers (Elmeer and Mattat 2012) Increasingly, 42 % of these loci were determined as homozygous while 58 % were heterozygous allelic loci. Similarly, according to Al-Dous et al. 2011, 3.5 million SNP genotypes were identified and scanned in the date palm genome for male and female polymorphism that segregates with sex. They examined that same heterozygous genotypes were shared by the male genomes whereas female genome shared the similar homozygous genotypes (Table 3).

**Table 3:** Details of date palm parents and progeny along their collection site

Sr. no.	Plants	Accession code	Collection site
1	Male	M-1 Hillawi	Sq. No-9, UAF
2	Male	M-2 Unknown	Sq. No-9, UAF
3	Male	M-3 Unknown	Sq. No-9, UAF
4	Male	M-4 Unknown	Sq. No-9, UAF
5	Male	M-5 Khudrawi	Sq. No-9, UAF
6	Male	M-6 Kurai	Dera Ismail Khan
7	Male	M-7 Unknown	University Campus
8	Male	M-8 Unknown	Sq. No-32, UAF
9	Female	H-1 Hillawi	Sq. No-32, UAF
10	Female	H-2 Hillawi	Sq. No-32, UAF
11	Female	H-3 Hillawi	Sq. No-32, UAF
12	Female	K-1 Khudrawi	Sq. No-32, UAF
13	Female	K-2 Khudrawi	Sq. No-32, UAF
14	Female	K-3 Khudrawi	Sq. No-32, UAF
15	Progeny	HM-1	Sq. No-32, UAF
16	Progeny	HM-2	Sq. No-32, UAF
17	Progeny	HM-3	Sq. No-32, UAF
18	Progeny	HM-4	Sq. No-32, UAF
19	Progeny	HM-5	Sq. No-32, UAF
20	Progeny	HM-6	Sq. No-32, UAF
21	Progeny	HM-7	Sq. No-32, UAF
22	Progeny	HM-8	Sq. No-32, UAF
23	Progeny	KM-1	Sq. No-32, UAF
24	Progeny	KM-2	Sq. No-32, UAF
25	Progeny	KM-3	Sq. No-32, UAF
26	Progeny	KM-4	Sq. No-32, UAF
27	Progeny	KM-5	Sq. No-32, UAF
28	Progeny	KM-6	Sq. No-32, UAF
29	Progeny	KM-7	Sq. No-32, UAF
30	Progeny	KM-8	Sq. No-32, UAF

Primer mpdCIR48 produced a specific locus (250/250) in all male plants whereas the same locus was absent in female plants. Primer mpdCIR25 identified six male plants with a specific homozygous locus 270/270. Similarly primer mpdCIR10 amplified two different fragments, one homozygous locus (230/230 bp) in male-2, while one heterozygous locus (230/240) in male-4 and male-5. The marker mpdCIR16 scored one homozygous fragment (190/190 bp) in male-1 and another fragment of 180/180 bp was identified in male-3. Primer mpdCIR25 characterized six male plants out of eight and a locus of 270/270 bp was present in male samples of 1, 2, 4, 5, 7, 8. Likewise, mpdCIR32 amplified a specific fragment 290/294 bp in three male samples. Three heterozygous loci were scored in male-4, 5 and 6 while a single homozygous locus (235/235 bp) was only amplified in male-8.

**Fig. 1:**
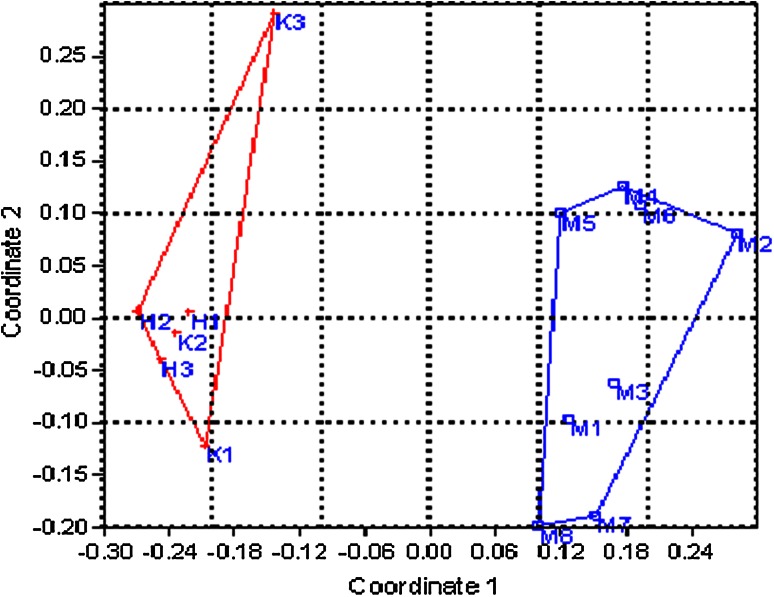
Scatter plot of first and second principle coordinate analysis (PCoA) of male and female parents based on the SSR from 12 SSR primers

**Fig. 2:**
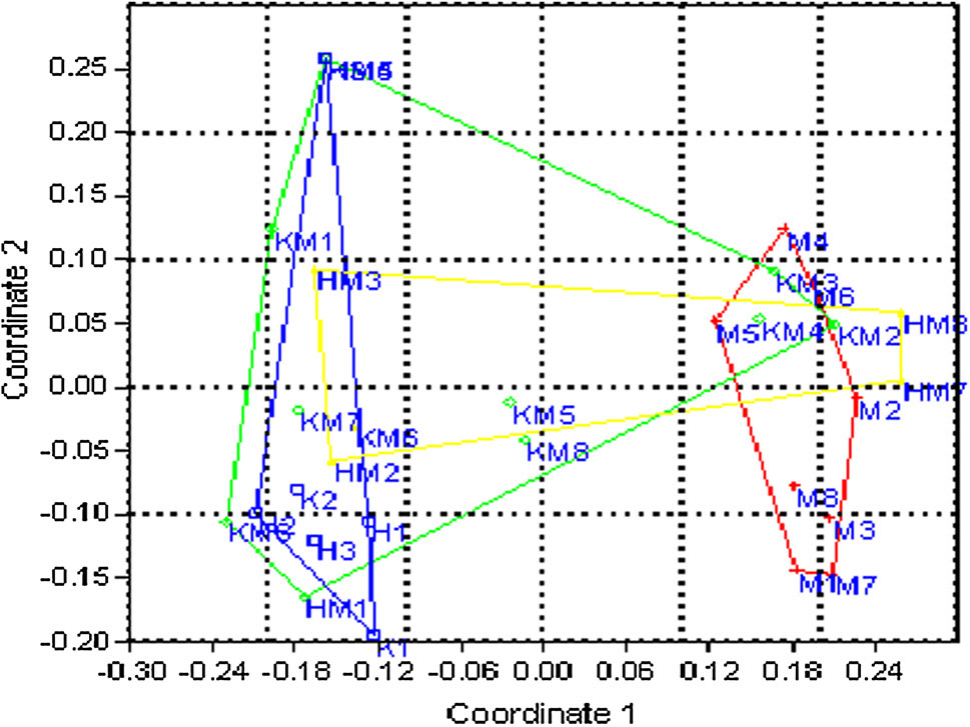
Scatter plot of first and second principle coordinate analysis (PCoA) using hamming distance measures in convex hulls based on the SSR from 12 primers

Great variations were observed in primer mpdCIR57, where a locus of 85/85 bp was found in male-3 and male-4 and a fragment of 295/295 bp was found in male-2 that is the most promising locus to determine the male plants from the progeny. Moreover, primer mpdCIR70 differentiated the two male plants with a heterozygous locus 300/310 bp and a similar locus was identified in male-2, 3, 4, 5 and male-6 with the primer DP-168. Following two allele sized 170/175 bp amplified with primer mpdCIR 93 and 160/162 bp (exhibited by primer mpdCIR93) were repeated twice in male 4 and 7, but these alleles were not observed in six female date palm trees. The data resulted from combination of 12 primers enabled the seedling samples of date palm cultivars to be divided into two groups regarding their sex expression (male and female) compared to their parents using the principle coordinate analysis (PCoA). Preferably Hamming distance was selected because, for shared characteristics it does not consider the common absence of alleles. For present study it was therefore considered to be most suitable, that included highly polymorphic data of microsatellite straddling policy at two levels. The PCoA suggested the total four broad groups comprising 8 male parents, 6 female parents, 11 male progenies and 5 female progenies. Trees are autonomous with 29 % variations explained by first axis and 14 % by the second axis. Figures 1 and 2 depict the variation within the parents, among the parents and the progeny. These findings are in accordance with the findings of date palm in Qatar (Elmeer and Mattat 2012). Male and female parents comprised separate groups while grouping of progeny into male and female fall between two parent groups that showed the sharing of alleles from both the parents and confirmed the true type hybridization. Male parents were found highly diverse from female parents and progeny. Among male parents, M8 and M5 were more divergent than other male plants, whereas the female sample number 20, 21 and 22 (H1, H2, H3) which belongs to same variety Hillawi, were closely compared with K1, K2, and K3 while K3 showed some genetic distances.

In date palm, limited numbers of suckers are produced, i.e., 15–20 throughout the female tree life, difficult to handle the large population, tedious and costly, need space and resources, so farmers are currently facing the problem to propagate from seeds. But farmers are unable to identify the gender of the date palm trees until it reaches the reproductive age (5–10 years) as this species is very slow flowering. The use of the flowering and vegetative characters (Rhouma 1994) and isozymes markers (Salem et al. 2001) are less productive as it takes a long time to evidence. So, fortunately SSR markers were used for molecular discrimination for unlimited date palm cultivars. This study is more precise and accurate compared to other date palm studies, i.e. plastid DNA halophytes (Sakka 2003) and isozymes (Salem et al. 2001; Booij et al. 1995). These data proved that SSR markers are more influential, powerful and key identifiers of date palm sex for speeding up the breeding programs.

## Conclusion

Present study is the first comprehensive investigation on the sex identification of date palm seedlings with SSR markers from Pakistan. The 1-year-old date palm seedlings were characterized as male and female, and identified candidate markers involved in sex determination. This study will provide a substantial resource for speeding up the date palm breeding programs. These results will be helpful for an efficient screening, management and use of date palm genetic resources in selection and breeding program.
